# Distinct Changes of BTLA and HVEM Expressions in Circulating CD4^+^ and CD8^+^ T Cells in Hepatocellular Carcinoma Patients

**DOI:** 10.1155/2018/4561571

**Published:** 2018-07-18

**Authors:** Jiayu Liu, Jiaqian Li, Min He, Geng-Lin Zhang, Qiyi Zhao

**Affiliations:** ^1^Department of Infectious Diseases, Third Affiliated Hospital, Sun Yat-sen University, Guangzhou, China; ^2^Guangdong Provincial Key Laboratory of Liver Disease, Third Affiliated Hospital, Sun Yat-sen University, Guangzhou, China; ^3^Guangdong Provincial Key Laboratory of Malignant Tumor Epigenetics and Gene Regulation, Medical Research Center, Sun Yat-Sen Memorial Hospital, Sun Yat-Sen University, Guangzhou, China; ^4^Breast Tumor Center, Sun Yat-Sen Memorial Hospital, Sun Yat-Sen University, Guangzhou, China; ^5^Department of Clinical Laboratory, Guangdong Provincial Hospital of Chinese Medicine, Guangzhou, China

## Abstract

BTLA/HVEM (B and T lymphocyte attenuator/herpes virus entry mediator) pathways play a critical role in T cell suppression in tumor. However, its dynamic changes in different T cell subsets in peripheral blood and their clinical significance are largely unclear in cancer patients. In the current study, we showed distinct changes of BTLA and HVEM expressions on peripheral blood CD4^+^ and CD8^+^ T cells in patients with hepatocellular carcinoma (HCC); BTLA expression were significantly upregulated on circulating CD4^+^ but not CD8^+^ T cells. In sharp contrast, the levels of HVEM expression were significantly downregulated on circulating CD8^+^ but not CD4^+^ T cells. A strong positive correlation between BTLA expression on circulating CD4^+^ T cells and BTLA expression on autologous CD8^+^ counterparts was observed in healthy donors but absent in HCC patients. More importantly, we found that blockade of the BTLA/HVEM pathway increased IFN-*γ* production in both circulating CD4^+^ and CD8^+^ T cells. Collectively, our data suggested that the BTLA/HVEM pathway contributes to peripheral T cell suppression in HCC patients, and BTLA/HVEM may serve as attractive targets for HCC immunotherapy.

## 1. Introduction

Hepatocellular carcinoma (HCC) is one of the most common malignancies worldwide, which rates as the third cause of cancer-related mortality [1, 2]. HCC is characterized by progressive development, high postsurgical recurrence, and extremely poor prognosis [1, 3]. Therefore, there is a pressing need to explore the novel therapy to supplement the conventional HCC treatments [3–6]. Recently, agents targeting the coinhibitory molecules such as the PD-1/PD-L1 pathway have shown promising results in cancer clinical trials [4, 7–12], indicating a prospect that immunotherapy may play a crucial role in the effective treatments of various types of cancers, including HCC [13–20].

Beyond the PD-1/PD-L1 pathway, emerging evidences show that the BTLA/HVEM pathway (B and T lymphocyte attenuator/herpes virus entry mediator) plays a key role in T cell inhibition in tumor microenvironment [21–24]. BTLA could interact with its ligand HVEM in *cis* or in *trans* [25, 26]. Expression of BTLA and HVEM on the surface of the same cell provides the possibility of *cis* interactions [27, 28]. The *cis* interaction between BTLA and HVEM is the predominant form expressed on the surface of naive human T cells, helping to maintain T cells in the naive state [25, 27]. By comparison, *trans* interactions occur between BTLA expressed on the surface of one cell and HVEM expressed on the surface of a separate cell, inducing inhibitory signaling via ITIM motifs [26, 28]. Although the important role of BTLA in peripheral tolerance induction of both CD4^+^ and CD8^+^ T cells has been appreciated by using BTLA-deficient mice [29, 30], the expression and function of BTLA on CD4^+^ and CD8^+^ T cells in human diseases remain elusive. In the recent study, we observed a significantly increased BTLA expression on tumor-infiltrating CD4^+^ T cells in HCC patients [31]. The interaction of BTLA and HVEM suppressed cytokine production of tumor-infiltrating CD4^+^ T cell [31]. However, the dynamic changes and role of BTLA and HVEM on peripheral blood T cells in patients with HCC remain unknown.

In the current study, by comparing the circulating T cells of HCC patients to the ones of healthy donors, we found that BTLA expressions of CD4^+^ T cells but not CD8^+^ T cells were significantly upregulated in HCC patients, whereas HVEM levels were significantly downregulated on circulating CD8^+^ rather than CD4^+^ T cells. Blockade of the BTLA/HVEM pathway increased IFN-*γ* production in both circulating CD4^+^ and CD8^+^ T cells. Collectively, our study suggests that these distinct changes of BTLA and HVEM expressions on circulating CD4^+^ and CD8^+^ T cell contribute to immunosuppressive state of HCC patients and may serve as attractive targets for HCC immunotherapy.

## 2. Patients and Methods

### 2.1. Patients and Specimens

37 patients with HCC underwent curative resection between 2014 and 2015 in the Third Affiliated Hospital of Sun Yat-sen University, and blood samples were collected and were used for the isolation of peripheral blood lymphocytes. Among them, peripheral blood lymphocytes from 25 patients were used for surface marker evaluation once isolated; peripheral blood lymphocytes from the other 12 patients were used for intracellular cytokine evaluation after culture (Table 1). None of the patients had received anticancer therapy before the sampling, and individuals with concurrent autoimmune diseases, HIV, or syphilis were excluded. Clinical stages were classified according to the guidelines of the International Union Against Cancer. As controls, 26 healthy donors who never had any form of neoplastic disease were recruited from the Third Affiliated Hospital of Sun Yat-sen University (Table 1). All samples were anonymously coded in accordance with local ethical guidelines (as stipulated by the Declaration of Helsinki), and written informed consent was obtained. The protocol was approved by the Review Board of the Third Affiliated Hospital of Sun Yat-sen University.

### 2.2. Isolation of Lymphocytes from Peripheral Blood

Peripheral blood lymphocytes were isolated from the blood of HCC patients and healthy donors by Ficoll density gradient centrifugation [32, 33] and then used for flow cytometry analysis or cell culture.

### 2.3. Cell Culture and Blocking Assay

Peripheral blood lymphocytes were cultured in RPMI1640 supplied with 10 IU/mL IL-2 (eBioscience). In some experiments, cells were pretreated with a blocking Ab against BTLA (eBioscience) or a control IgG (R&D Systems).

### 2.4. Flow Cytometry

Peripheral blood lymphocytes were stained with fluorochrome-conjugated mAbs for CD3, CD4, CD8, HVEM, IFN-*γ* (eBioscience), BTLA (BD Pharmingen), or control Ab (eBioscience). Lymphocytes were left untreated, or in some experiments, stimulated at 37°C for 5 h with Leukocyte Activation Cocktail (BD Pharmingen) [34]. Thereafter, cells were stained with surface markers, fixed and permeabilized with intraPrep reagents (Beckman Coulter), and finally stained with intracellular markers. Data were acquired on a Gallios flow cytometer (Beckman Coulter) and analyzed with FlowJo software. CD4^+^ and CD8^+^ T cells were gated based on CD3 and CD4 or CD8 expression, respectively [35]. Samples costained with CD3/CD4/CD8/BTLA or CD3/CD4/CD8/HVEM were used to investigate the correlation of the BTLA or HVEM level between CD4^+^ and CD8^+^ T cells.

### 2.5. Statistical Analysis

All data were analyzed using the SPSS version 13.0 software (Chicago, IL, USA) and summarized as means and standard errors or median and range. Correlation analysis was evaluated by the Spearman's rank correlation test. Statistical significance was determined by Student's *t*-test. All data were analyzed using 2-tailed tests unless otherwise specified, and a *P* value of less than 0.05 was considered statistically significant.

## 3. Results

### 3.1. BTLA Expression on Circulating CD4^+^ but Not CD8^+^ T Cells Were Significantly Upregulated in HCC Patients

**Figure 1:**
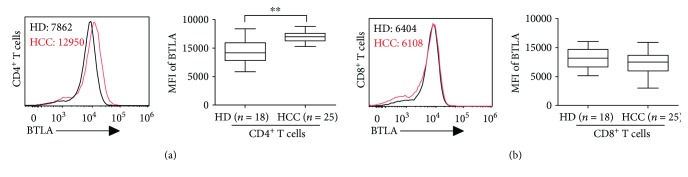
Expression of BTLA on peripheral CD4^+^ and CD8^+^ T cells in HCC patients. (a) BTLA expression on CD4^+^ T cells from HCC patients (red line) and healthy donors (HD, black line) was determined by flow cytometry, and median mean florescence (MFI) was indicated (left panel). Box plots of MFI of BTLA on CD4^+^ T cells from HCC patients (*n* = 25) and HD (*n* = 18) were shown in the right panel. (b) Expressions of BTLA on CD8^+^ T cells were displayed as in (a). ^∗∗^*P* < 0.01.

We previously showed that increased BTLA expression on tumor-infiltrating CD4^+^ T cells contributes to T cell suppression in tumor in situ [31]. To evaluate the potential role of BTLA in circulating T cells, we examined surface expression of BTLA on circulating T cells freshly isolated from 25 HCC patients and 18 healthy donors (HD). In the samples analyzed, BTLA was highly expressed on circulating CD4^+^ T cells in HD, with more than 90% positive rate (Figure 1(a)). As expected, we observed a significantly increased BTLA expression on CD4^+^ T cells in peripheral blood from HCC patients, up to 31% increase measured by mean florescence intensity (MFI) as compared to that of HD (median MFI: 12042 versus 9195, *P* < 0.01, Figure 1(a)).

Surprisingly, we did not observe significant difference of BTLA expression on circulating CD8^+^ T cells in HCC patients as compared to that of HD (Figure 1(b)). Collectively, our results show that BTLA expressions were upregulated on circulating CD4^+^ but not CD8^+^ T cells in HCC patients.

### 3.2. HVEM Expression on Circulating CD8^+^ but Not CD4^+^ T Cells Were Significantly Downregulated in HCC Patients

**Figure 2:**
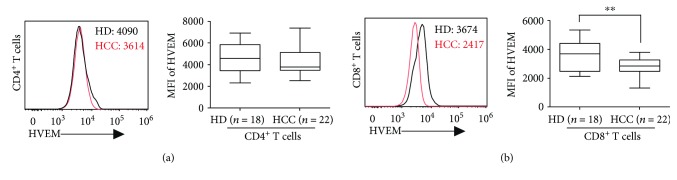
Expression of HVEM on peripheral CD4^+^ and CD8^+^ T cells in HCC patients. (a) HVEM expression on CD4^+^ T cells from HCC patients (red line) and healthy donors (HD, black line) was determined by flow cytometry, and median mean florescence (MFI) was indicated (left panel). Box plots of MFI of HVEM on CD4^+^ T cells from HCC patients (*n* = 22) and HD (*n* = 18) were shown in the right panel. (b) Expressions of HVEM expression on CD8^+^ T cells were displayed as in (a). ^∗∗^*P* < 0.01.

The functional outcome of BTLA engagement is complicated by its widespread and varied interaction pattern [25]. Therefore, we examined surface expression of HVEM, the ligand for BTLA [30], on circulating T cells freshly isolated from 22 HCC patients and 18 healthy donors (HD). In the samples analyzed, HVEM was highly expressed on circulating CD4^+^ T cells in HD, with more than 98% positive rate and median MFI up to 4567 (Figure 2(a)). In contrast to the change of BTLA expression on circulating CD4^+^ T cells in HCC patients, we did not observe significant difference of HVEM expression on circulating CD4^+^ T cells in HCC patients as compared to that of HD (Figure 2(a)).

Instead, we observed a significantly decreased HVEM expression on CD8^+^ T cells in peripheral blood from HCC patients, up to 29% decrease measured by MFI as compared to that of HD (median MFI: 2830 versus 3647, *P* < 0.01, Figure 2(b)). Collectively, our results show that HVEM expression was downregulated on circulating CD8^+^ but not CD4^+^ T cells in HCC patients.

### 3.3. BTLA and HVEM Levels Were Positively Correlated between Circulating CD4^+^ and CD8^+^ T Cells

**Figure 3:**
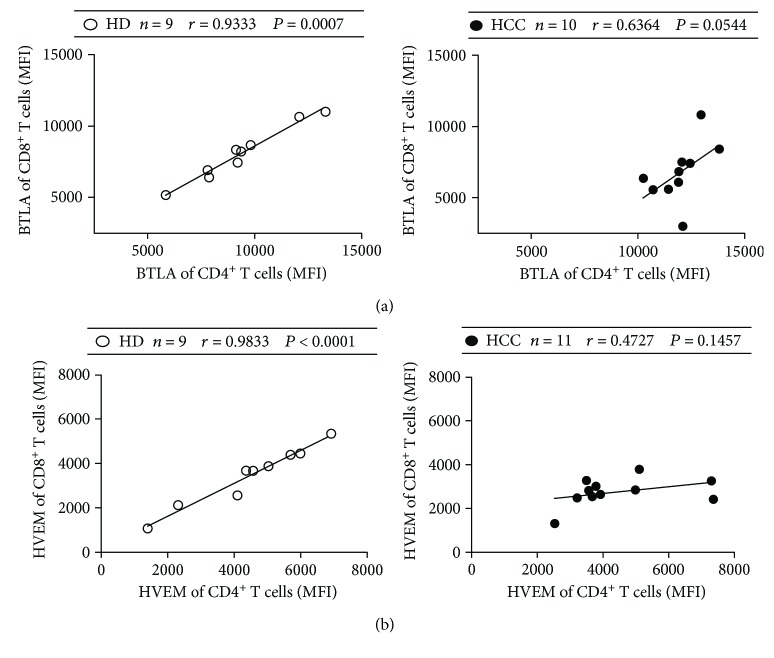
Correlations of BTLA/HVEM expression between peripheral CD4^+^ and CD8^+^ T cells. (a) Correlation of BTLA expression between peripheral CD4^+^ and CD8^+^ T cells in healthy donors (HD, open circle) and HCC patients (filled circle). (b) Correlation of HVEM expression between peripheral CD4^+^ and CD8^+^ T cells in healthy donors and HCC patients. Solid line: linear growth trend; *r*: correlation coefficient. *P* values are shown.

We next examined the correlation between the BTLA level on circulating CD4^+^ T cells and the BTLA level on circulating CD8^+^ T cells, as well as correlation of the HVEM level. A strong positive correlation existed between BTLA expression on circulating CD4^+^ and CD8^+^ T cells from healthy donors (*r* = 0.9333, *P* = 0.0007, Figure 3(a)). However, further analysis showed that such correlation was much weaker in HCC patients (*r* = 0.6364, *P* = 0.0544, Figure 3(a)). Similarly, a strong positive correlation existed between HVEM expression on circulating CD4^+^ and CD8^+^ T cells in healthy donors (*r* = 0.9833, *P* < 0.0001, Figure 3(b)) but not in HCC patients (*r* = 0.4727, *P* = 0.1457, Figure 3(b)). These data indicate that although expressions of BTLA and HVEM between circulating CD4^+^ and CD8^+^ T cells were strongly correlated in the steady state as shown in healthy donors, the changes of BTLA and HVEM expressions between circulating CD4^+^ and CD8^+^ T cells might reflect an imbalanced immune state in HCC patients.

### 3.4. BTLA/HVEM Pathway Suppressed IFN-*γ* Production in Both Circulating CD4^+^ and CD8^+^ T Cells in HCC Patients

**Figure 4:**
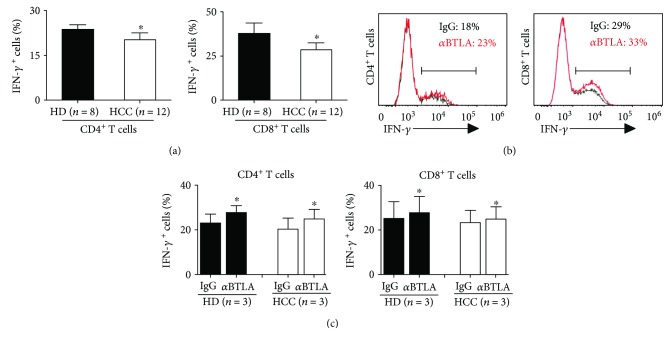
Blockade of the BTLA increased the frequency of cytokine-producing CD4^+^ and CD8^+^ T cells. (a) The capacity of circulating CD4^+^ and CD8^+^ T cell subsets to produce IFN-*γ* ex vivo (*n* = 12 for HCC, *n* = 8 for HD). (b and c) Peripheral blood lymphocytes from HCC patients and healthy donors were incubated overnight with anti-BTLA mAb (*α*BTLA, red line) or an isotype control antibody (IgG, black line) before evaluating intracellular cytokine production (*n* = 3 for HCC, *n* = 3 for HD). The gates showed the frequency of IFN-*γ*-producing cells among circulating CD4^+^ or CD8^+^ T cells (b). Results are expressed as mean ± SEM. ^∗^*P* < 0.05.

Both circulating CD4^+^ and CD8^+^ T cells from HCC patients exhibited reduced capacity to produce IFN-*γ* (*P* < 0.05 compared with those from HD, *n* = 12, Figure 4(a)). To determine whether BTLA/HVEM pathway blockade improved the IFN-*γ* production of the circulating T cells, we isolated peripheral blood lymphocytes and cultured them in the presence of anti-BTLA blocking antibodies or control IgG. Treatment with anti-BTLA resulted in a higher percentage of IFN-*γ* production in both CD4^+^ and CD8^+^ T cells (*n* = 3, Figures 4(b) and 4(c)). These data suggests that the BTLA pathway might contribute to peripheral CD4^+^ and CD8^+^ T cell suppression in HCC patients.

## 4. Discussion

The BTLA/HVEM pathway has been identified as a critical regulator in tumor immunity [25, 36, 37], but its dynamic changes in peripheral blood T cell subsets and their clinical significance are largely unclear in human malignancies [37]. Here, we showed that changes of BTLA and HVEM expressions in peripheral blood CD4^+^ and CD8^+^ T cells from HCC patients were different; BTLA expression was significantly upregulated on circulating CD4^+^ but not CD8^+^ T cell subset, while HVEM expression was significantly downregulated on circulating CD8^+^ but not CD4^+^ T cell subset. Of note, blockade of the BTLA/HVEM pathway increased IFN-*γ* production in both circulating CD4^+^ and CD8^+^ T cell subset. Collectively, our findings suggest that distinct changes of BTLA and HVEM expressions in circulating CD4^+^ and CD8^+^ T cells might play a role in immune suppression of HCC patients.

**Figure 5:**
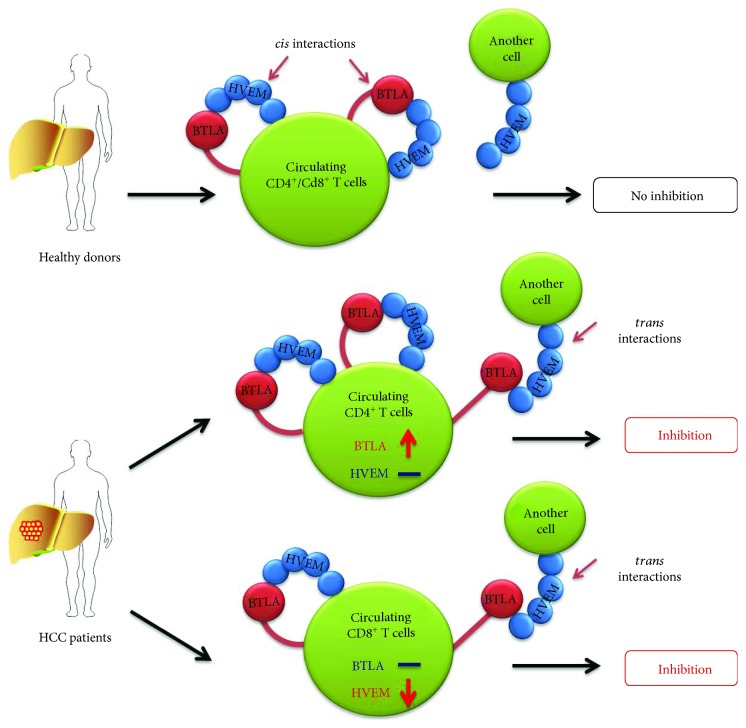
Proposed model for distinct changes of BTLA and HVEM in circulating CD4^+^ and CD8^+^ T cells. On circulating CD4^+^ and CD8^+^ T cells from healthy donors, the *cis* interaction between BTLA and HVEM is the predominant form, helping to maintain T cells in the naive state. In HCC patients, BTLA expressions were significantly upregulated while HVEM expression remained unchanged on circulating CD4^+^ T cells. In sharp contrast, HVEM expressions were significantly downregulated while BTLA expression remained unchanged on circulating CD8^+^ T cells. Both provide increased inhibitory signaling through BTLA engaged by intercellular HVEM in *trans*.

As an important axis of coinhibitory receptor/ligand, the BTLA/HVEM pathway exerts considerable effect on keeping T cell from excessive activation and regulating T cell suppression in healthy individuals [38–41]. Our findings suggest that distinct changes of BTLA and HVEM expressions on circulating CD4^+^ and CD8^+^ T cells might together lead to immune suppression of HCC patients (Figure 5). First, BTLA engagement by HVEM expressed on a separate cell in *trans* could mediate inhibitory signaling via ITIM motifs [25, 26]. Our data showed that BTLA expressions were significantly upregulated while HVEM expression remained unchanged on circulating CD4^+^ T cells in HCC patients compared to that in healthy donors (Figures 1(a) and 2(a)), providing an increase of inhibitory signaling through BTLA engaged by intercellular HVEM in *trans*. Second, BTLA could interact with HVEM in *cis* on T cells in which both BTLA and HVEM are expressed, which alleviate coinhibitory signaling by BTLA [26, 27]. Our data showed that HVEM expression was significantly downregulated on circulating CD8^+^ T cells in HCC patients, while appreciable difference in BTLA expression was absent (Figures 1(b) and 2(b)). Such net reduction of HVEM on circulating CD8^+^ T cells might increase the coinhibitory signaling by disassociated BTLA molecules from the HVEM-BTLA *cis* complex [26]. Taken together, our data show that selective BTLA overexpression on circulating CD4^+^ T cells and HVEM suppression on circulating CD8^+^ T cells lead to increased coinhibitory signaling by BTLA, which contributes to peripheral T cell suppression in HCC patients. In addition, we found that blockade of BTLA signaling increased IFN-*γ* production in both circulating CD4^+^ and CD8^+^ T cells isolated from HCC patients (Figure 4), which strengthens the emerging notion that BTLA can be used as a target of cancer immunotherapy [42]. Consistently, our previous studies demonstrated that BTLA expression significantly increased on tumor-infiltrating CD4^+^ T cells which suppressed cytokine production in HCC patients [31]. Therefore, we and other studies established that the BTLA/HVEM pathway on both circulating T cells and tumor-infiltrating T cells plays a key role in tumor immunosuppression and serves as potential targets for cancer immunotherapy [14, 25, 31, 37, 39, 42, 43].

The expression patterns of coinhibitory receptors were distinct for CD4^+^ and CD8^+^ T cells [44, 45]. In the current study, we observed marked upregulation of BTLA expression on circulating CD4^+^ but not CD8^+^ T cells (Figures 1(a) and 2(a)), which is in consistent with our previous studies on BTLA in tumor-infiltrating T cells in HCC patients [31]. We recently reported that HVEM levels were downregulated on peripheral CD3^+^ T cells in HCC patients [43]. On this basis, our current study further showed that decreased HVEM levels on CD3^+^ T cells were mainly due to decreased HVEM levels on CD8^+^ T cell subset (Figures 1(b) and 2(b)). Such distinct changes of BTLA and HVEM expressions in tumor patients were attributed to the absence of their positive correlation between CD4^+^ and CD8^+^ T cells, which can be observed in healthy donors (Figure 3). Recent studies in the lymphocytic choriomeningitis virus (LCMV) mouse model have directly compared the transcription programs of exhausted CD4^+^ and CD8^+^ T cells [44, 46]. Our studies extend the current understanding about a distinct coinhibitory molecule expression pattern in CD4^+^ and CD8^+^ T cells, by showing distinct changes of BTLA and HVEM expressions on CD4^+^ and CD8^+^ T cell subset in human cancers. Exploring the underlying mechanism of the distinctive expression pattern of BTLA and HVEM in different T cell subsets is an important future goal for designing more effective cancer immunotherapy.

In summary, our studies suggest that the BTLA/HVEM pathway might contribute to peripheral T cell suppression in HCC patients. The distinct changes of BTLA and HVEM on circulating CD4^+^ and CD8^+^ T cells could be taken into consideration when targeting BTLA or HVEM in cancer immunotherapy [42, 47, 48]. Future studies are warranted to further explore the translational values of the BTLA/HVEM pathway as therapeutic targets in various tumor types [15, 36, 49].

## Figures and Tables

**Table 1:** Demographical and clinical characteristics.

Characteristics	Cases for Figures 1–3		Cases for Figure 4	
	Patients	HD	Patients	HD
Number	25	18	12	8
Age, years (median, range)	48, 35–69	40, 25–52	45, 35–50	46, 40–55
Gender (male/female)	23/2	17/1	12/0	8/0
HBsAg (negative/positive)	1/24	18/0	0/12	8/0
Cirrhosis (absent/present)	7/18	—	3/9	—
ALT, U/L (median, range)	34, 16–81	—	39, 20–76	—
AFP, ng/mL (≤25/>25)	7/18	—	4/8	—
Tumor size, cm (≤5/>5)	15/22	—	15/22	—
Tumor multiplicity (solitary/multiple)	22/3	—	10/2	—
Vascular invasion (absent/present)	22/3	—	12/0	—
Intrahepatic metastasis (no/yes)	24/1	—	12/0	—
TNM stage (I + II/III + IV)	19/6	—	10/2	—
Tumor differentiation (I + II/III + IV)	18/7	—	9/3	—
Fibrous capsule (absent/present)	5/20	—	2/10	—

Abbreviations: HD: healthy donors; HBsAg: hepatitis B surface antigen; ALT: alanine aminotransferase; AFP: *α*-fetoprotein; TNM: tumor node metastasis.

## Data Availability

Data supporting this study are provided in the Results and Patients and Methods' sections in this paper. The data used or analyzed in the current study are available from the corresponding author (zhaoqyi@mail.sysu.edu.cn) on reasonable request.

## Abbreviations

BTLA: B and T lymphocyte attenuator
HCC: Hepatocellular carcinoma
HVEM: Herpes virus entry mediator
MFI: Mean florescence intensity
TNM: Tumor node metastasis.
